# Language Disparities in Continuous Glucose Monitoring for Type 2 Diabetes

**DOI:** 10.1001/jamanetworkopen.2025.16523

**Published:** 2025-06-17

**Authors:** Jorge A. Rodriguez, Nicolás Prada-Rey, Chenxi Gao, Wenyu Song, Stuart R. Lipsitz, Courtney R. Lyles, A. Enrique Caballero, David W. Bates, Lipika Samal

**Affiliations:** 1 Division of General Internal Medicine and Primary Care, Brigham and Women’s Hospital, Boston, Massachusetts; 2 Harvard Medical School, Boston, Massachusetts; 3 Center for Healthcare Policy and Research, University of California Davis, Davis; 4 Department of Public Health Sciences, University of California Davis School of Medicine, Davis

## Abstract

This cross-sectional study examines the association between language preference and receipt of continuous glucose monitor prescription among adults with type 2 diabetes.

## Introduction

Among the 34.1 million US adults with type 2 diabetes (T2D), those with non–English language preference (NELP) face worse health outcomes.^1,2^ Continuous glucose monitors (CGMs) are patient-facing devices providing real-time glucose data. CGMs are associated with improved hemoglobin A_1c_ (HbA_1c_) levels and decreased diabetes-related health care use compared with traditional glucose monitoring.^3^ While prior studies have documented inequitable CGM access, the role of language preference in CGM access remains unexplored.^4^ This study aimed to compare CGM prescriptions between patients with NELP and patients with English language preference (ELP).

## Methods

The cohort consisted of adults with T2D receiving primary care or endocrinology care at a large integrated health care system from January 1, 2022, to December 31, 2023. Patients with T2D were identified using a validated electronic health record (EHR)–based algorithm.^5^ Language preference was dichotomized as NELP or ELP according to the EHR’s preferred language field. The Brigham and Women’s Hospital Institutional Review Board approved this cross-sectional study and waived informed consent because deidentified data were used. We followed the STROBE reporting guideline.

The primary outcome was CGM prescription (any CGM order in EHR) placed before December 31, 2023. Covariates included demographic characteristics (age, sex, and race and ethnicity), insurance type, clinical factors (insulin use and diabetes control [HbA_1c_ ≤9% vs >9%]), and care type (primary care only, endocrinology care only, or both). We conducted sensitivity analyses restricted to patients with uncontrolled diabetes (HbA_1c_ >9%) or patients using insulin. We performed subgroup analyses of patients with NELP, comparing them by Spanish vs non–Spanish language preference. Descriptive statistics were used to examine patient characteristics, with χ^2^ for categorical variables and Wilcoxon rank sum for continuous variables. Multivariable logistic regression including all covariates was used for adjusted analyses. We also estimated adjusted estimated probabilities and average marginal effects. A complete case analysis was performed.

Two-sided *P* < .05 indicated statistical significance. Analyses were performed in R 4.4.0 (R Project for Statistical Computing).

## Results

The study included 69 269 patients with T2D (median [IQR] age, 60 [51-69] years; 34 958 males [50.5%]), of whom 43.5% had public insurance. Patients with NELP composed 12.1% of the cohort. Compared with patients with ELP, patients with NELP were older and more likely to be female, have Hispanic ethnicity, have public insurance, use insulin, and have uncontrolled diabetes but less likely to see endocrinologists (Table 1). Overall, 12.2% of patients had at least 1 CGM prescription.

**Table 1.** Characteristics of Patients With Type 2 Diabetes by Language Preference

Characteristic	Main cohort, No. (%)			*P* value	Subgroup of patients with NELP, No. (%)		*P* value
	Overall (n = 69 269)	ELP (n = 60 895)	NELP (n = 8374)		Spanish language preference (n = 5063)	Non–Spanish language preference (n = 3311)	
Age, median (IQR), y	60 (51-69)	60 (51-68)	61 (52-70)	<.001	59 (50-67)	65 (56-74)	<.001
Sex							
Female	34 311 (49.5)	29 213 (48.0)	5098 (60.9)	<.001	3166 (62.5)	1932 (58.4)	<.001
Male	34 958 (50.5)	31 682 (52.0)	3276 (39.1)		1379 (41.6)	1897 (37.5)	
Race and ethnicity^a^							
Asian, non-Hispanic	4693 (6.8)	3604 (5.9)	1089 (13.0)	<.001	1 (<0.1)	1088 (32.9)	<.001
Black, non-Hispanic	6320 (9.1)	5784 (9.5)	536 (6.4)		13 (0.3)	523 (15.8)	
Hispanic	8370 (12.1)	3518 (5.8)	4852 (57.9)		4773 (94.3)	79 (2.4)	
White, non-Hispanic	48 487 (70.0)	47 009 (77.2)	1478 (17.6)		78 (1.5)	1400 (42.3)	
Other, non-Hispanic^b^	1399 (2.0)	980 (1.6)	419 (5.0)		198 (3.9)	221 (6.7)	
Insurance type							
Private	35 377 (51.1)	31 472 (51.7)	3905 (46.6)	<.001	2425 (47.9)	1480 (44.7)	<.001
Public	30 128 (43.5)	26 147 (42.9)	3981 (47.5)		2384 (47.1)	1597 (48.2)	
Other^c^	3764 (5.4)	3276 (5.4)	488 (5.8)		254 (5.0)	234 (7.1)	
Insulin use							
No	56 840 (82.1)	50 127 (82.3)	6713 (80.2)	<.001	3972 (78.5)	2741 (82.8)	<.001
Yes	12 429 (17.9)	10 768 (17.7)	1661 (19.8)		1091 (21.5)	570 (17.2)	
Diabetes control							
HbA_1c_ ≤9%	64 249 (92.8)	56 761 (93.2)	7488 (89.4)	<.001	4456 (88.0)	3032 (91.6)	<.001
HbA_1c_ >9%	5020 (7.2)	4134 (6.8)	886 (10.6)		607 (12.0)	279 (8.4)	
Care type							
Both endocrine care and primary care	13 715 (19.8)	12 423 (20.4)	1292 (15.4)	<.001	764 (15.1)	528 (15.9)	.39
Endocrine care only	4444 (6.4)	3942 (6.5)	502 (6.0)		315 (6.2)	187 (5.6)	
Primary care only	51 110 (73.8)	44 530 (73.1)	6580 (78.6)		3984 (78.7)	2596 (78.4)	

Abbreviations: ELP, English language preference; HbA_1c_, hemoglobin A_1c_; NELP, non–English language preference.

SI conversion factor: To convert the percentage of total hemoglobin to the proportion of total hemoglobin, multiply by 0.01.

^a^ Race and ethnicity were self-reported, assessed during patient registration, and extracted from the electronic health record. These demographic factors were included based on prior literature documenting disparities in continuous glucose monitor use across racial and ethnic groups. The initial dataset included the following race options: American Indian or Alaska Native, Asian, Black or African American, Native Hawaiian or Other Pacific Islander, White, and other (information on other category could not be accessed).

^b^ In the present study, this category includes non-Hispanic American Indian or Alaska Native, Native Hawaiian or Other Pacific Islander, and other race and ethnicity.

^c^ Information on other insurance types could not be accessed.

In unadjusted analyses, a lower proportion of patients with NELP had a CGM prescription compared with patients with ELP (7.4% vs 12.7%; *P* < .001). In adjusted analyses, patients with NELP were significantly less likely to have a CGM prescription (adjusted odds ratio [AOR], 0.58; 95% CI, 0.52-0.65; *P* < .001) (Table 2). This difference persisted in sensitivity analyses of patients with uncontrolled diabetes (AOR, 0.59; 95% CI, 0.46-0.75; *P* < .001) and patients using insulin (AOR, 0.70; 95% CI, 0.60-0.81; *P* < .001).

**Table 2.** Association Between Language Preference and Continuous Glucose Monitor Prescription

Analysis	Unadjusted OR (95% CI)	*P* value	Adjusted OR (95% CI)	*P* value	Adjusted predicted probabilities (95% CI), %	Average marginal effect (95% CI), %
Primary analysis (N = 69 269)^a^						
NELP	0.54 (0.50 to 0.59)	<.001	0.58 (0.52 to 0.65)	<.001	8.5 (7.8 to 9.2)	−4.1 (−4.8 to −3.3)
ELP	1 [Reference]		1 [Reference]		12.6 (12.4 to 12.9)	
Sensitivity analyses^a^						
Insulin users only (n = 5020)						
NELP	0.57 (0.50 to 0.63)	<.001	0.70 (0.60 to 0.81)	<.001	31.5 (29.0 to 34.3)	−7.48 (−10.4 to −4.5)
ELP	1 [Reference]		1 [Reference]		39.1 (38.2 to 40.0)	
HbA_1c_ >9% only (n = 12 429)						
NELP	0.51 (0.42 to 0.62)	<.001	0.59 (0.46 to 0.75)	<.001	19.6 (16.6 to 22.5)	−7.9 (−11.3 to −4.5)
ELP	1 [Reference]		1 [Reference]		27.4 (26.1 to 28.7)	
Subgroup analysis (NELP group only) (n = 8374)^b^						
Spanish language preference	1.08 (0.91 to 1.28)	.37	0.87 (0.72 to 1.06)	.17	7.1 (6.5 to 7.8)	−0.8 (−1.9 to 0.3)
Non-Spanish language preference	1 [Reference]		1 [Reference]		7.9 (7.0 to 8.8)	

Abbreviations: ELP, English language preference; HbA_1c_; hemoglobin A_1c_; NELP, non–English language preference; OR, odds ratio.

^a^ Adjusted for age, sex, race and ethnicity, insurance type, diabetes control (HbA_1c_ >9% vs ≤9%), insulin use, and care type.

^b^ Adjusted for age, sex, insurance type, diabetes control (HbA_1c_ >9% vs ≤9%), insulin use, and care type. Race and ethnicity were not used in this model due to collinearity.

In subgroup analyses, patients with Spanish language preference accounted for 60.5% of the subpopulation. They were younger and more likely to be female, have Hispanic ethnicity, have private insurance, use insulin, and have uncontrolled diabetes. There was no significant difference between patients with Spanish vs non–Spanish language preferences (AOR, 0.87; 95% CI, 0.72-1.06; *P* = .17).

## Discussion

We found disparities in CGM prescriptions by language preference, showing patients with NELP having less access to CGM. These findings align with those of previous research documenting racial and ethnic disparities in CGM access.^4^ Our study highlights the specific role of language preference in CGM prescribing patterns.

Study limitations include the single-site design, which may limit generalizability; our inability to assess whether CGM prescriptions translated to actual use; lack of data on clinician-patient language concordance; and unmeasured clinician preferences, which might affect CGM prescribing decisions. As CGM availability increases, there is an urgent need to identify and reduce barriers to equitable CGM adoption among patients with NELP.

## Notes

Supplement.
Data Sharing Statement
