# Supplementary material for: Language Disparities in Continuous Glucose Monitoring for Type 2 Diabetes
Source: JAMA Netw Open. 2025 Jun 17;8(6):e2516523. doi: 10.1001/jamanetworkopen.2025.16523 (PMC12175012; doi:10.1001/jamanetworkopen.2025.16523)
Supplement: Supplement. — Data Sharing Statement [file jamanetwopen-e2516523-s001.pdf]

Data Sharing Statement

Rodriguez. Language Disparities in Continuous Glucose Monitoring for Type 2 Diabetes. *JAMA Netw Open*. Published June 17, 2025. doi:10.1001/jamanetworkopen.2025.16523

Data
Data available: No

Additional Information
Explanation for why data not available: We will not be providing direct access to data to protect patient privacy. However, requests can be made to the authors for aggregate data.
